# Opportunistic Mental Health Screening: Is there a Role Following a Disaster? Lessons from the 2010-2011 Queensland (Australia) Floods and Cyclones

**DOI:** 10.1017/S1049023X23000092

**Published:** 2023-04

**Authors:** David Crompton, Peter Kohleis, Jane Shakespeare-Finch, Gerard FitzGerald, Ross Young

**Affiliations:** 1. Queensland University of Technology, Brisbane, Queensland, Australia; 2. Griffith University, Nathan, Queensland, Australia; 3. Metro South Hospital and Health Service, Woolloongabba, Queensland, Australia; 4. University Sunshine Coast, Maroochydore DC, Queensland, Australia

**Keywords:** natural disaster, opportunistic mental health screening, general health call line

## Abstract

**Background:**

Following the 2010-2011 floods and cyclones that affected 78% of Queensland, Australia, a State-wide mental health response was established. The response plan included a 24-hour access line. This study examines the effectiveness of the mental health screening program conducted via the State-wide health call center (13HEALTH) in 2012.

**Methods:**

Callers to the 13HEALTH line were screened to assess the impact of the disaster. The 13HEALTH clinicians administered the Primary Care-Posttraumatic Stress Disorder Scale (PC-PTSD) screening measure. Those scoring more than two on the PC-PTSD Scale were provided information on the emotional impact of disasters and a referral to the post-disaster specialist mental health program (SMHP). For calls related to those under 18, a single-item question assessed behavioral or emotional changes since the natural disasters. Those with identified changes were offered a referral to a post-disaster SMHP.

The study evaluates the relationship between disaster exposure and the likelihood of 13HEALTH callers experiencing physical health concerns and unacknowledged mental health symptoms. The program’s cost for the 12 months of 2012 was assessed using data from the financial contract.

**Results:**

In 2012, there were 205,064 calls to 13HEALTH: 19,708 identified as residing in a flood or cyclone-affected area, 7,315 adults indicated they were personally affected, and 907 scored more than two on the PC-PTSD Scale. Only 700 agreed to a referral to the SMHP. There were 290 children under 18 assessed as at risk; 207 accepted a referral to a SMHP.

Regions that experienced a greater impact from the floods and cyclones were 1.3-2.3 times more likely to report being personally affected by the floods and cyclones. Similarly, these regions had more callers scoring more than two on the PC-PTSD Scale. The total cost of the 13HEALTH program for 2012 was $53,284 (AU) across all age groups.

**Conclusion:**

The 13HEALTH general health post-disaster screening program demonstrates opportunistic screening may assist identification of those with unmet mental health needs. The data indicate an increased likelihood of personal exposure in the more affected regions with an increased risk of unrecognized psychological symptoms as assessed by the PC-PTSD Scale. However, more than 20% declined referral to a SMHP.

## Introduction

The July 2021 through March 2022 floods that affected urban and regional areas of Queensland and New South Wales^1,2^ Australia were, like the 2010-2011 Queensland floods and cyclones, associated with extensive social and community disruption.^3^ Some areas experienced multiple flooding episodes, with thousands of people evacuated. Media reports highlighted the emotional distress related to the number of deaths and the damage to businesses and homes.^4,5^

The psychosocial disruption following floods is well-documented, with the physical and psychological sequelae potentially lasting many years. It is recognized that the impact on urban and rural communities may differ.^6–10^ The World Health Organization (WHO; Geneva, Switzerland) predicts an increase in severe mental disorders in the twelve months after a disaster and a 10% to 20% increase in the incidence of depression and anxiety disorders.^11^ A disaster’s impact is not limited to the nature and severity of the event. Aside from exposure to the event, personal factors such as developmental trauma, pre-event health, and social and economic factors sway the psychosocial outcomes.^12–16^

The adverse psychosocial consequences of disasters have led to response frameworks that stress the critical role of government and non-government agencies, the inclusiveness of various disciplines, and the implementation of evidence-based psychological and psychiatric interventions.^17^ These interventions aim to address psychological disorders or distress that may emerge following the event, and those mental illnesses exacerbated by the disaster, and to reduce unmet mental health needs in affected communities.^18,19^

Australian post-disaster funding acknowledges the need for psychosocial support through specialized mental health programs, primary care, and community services.^20–22^ The need to address the immediate and long-term psychosocial impacts of the 2022 floods mirrors the demand for community and specialized mental health services following the Queensland disasters of 2010-2011. Following the events in 2010-2011, the Queensland Government implemented the Operation Queenslander Plan 2011-2013 (The Plan).^23^ The Plan included a State-wide specialist mental health program (SMHP) for those experiencing significant psychosocial distress. It emphasized the critical role of local service providers such as the public mental health services (PMHS), the non-government sector, general practitioners, and allied health practitioners. The Plan acknowledged many of those exposed to the disasters would not require specialist care and the demands on the PMHS often exceed available resources.^22,24^

The SMHP and a 24-hour access line were established to address the mental health needs of those experiencing a disaster-related mental illness and individuals considered at significant risk. The access line began as a service people contacted if they were concerned for their mental health following the floods and cyclones. As only five people called the service in six months, it was decided to move the program to 13HEALTH, an established, well-known, Queensland-wide 24-hour health line service; 13HEALTH provides telephone assessments and may link callers to primary care or one of the Queensland sixteen Hospital and Health Services (HSS).

The new program adopted an opportunistic screening strategy (Table 1). This approach required each person who rang 13HEALTH, usually due to a physical health concern, to be asked if they had been affected by the floods or cyclones. They were asked if they lived in a flood or cyclone-affected area and if they were agreeable to answering questions related to their emotional response to the floods and cyclones. Regardless of the screening results, each person was provided information on the services available to assist if they experienced distress.^22^

**Table 1.** 13HEALTH Screen for Effect of Floods and Cyclones 2010-2011

	Question	Score
Step 1	Did you or do you currently reside in a flood or cyclone-affected area?	Yes/No
Step 2	Were you personally affected by the floods or cyclones?	Yes/No
Step 3	In the past month, have you had nightmares about the flood/cyclone or thought about it when you did not want to?	No = 0 Yes = 1
	Have you tried hard not to think about it or gone out of your way to avoid situations that remind you of it?	No = 0 Yes = 1
	Have you been constantly on guard, watchful, or easily startled?	No = 0 Yes = 1
	Have you felt numb or detached from others, any activities, or your surroundings?	No = 0 Yes = 1
Step 4	**If score >2:** Thank you for answering these questions. You have noticed some changes in yourself since the floods and cyclones. There is a program called Recovery and Resilience; it offers families and individuals specialist advice and help concerning changes related to the floods and cyclones. I’m wondering if you would like someone from the program to contact you to talk about these changes. Would you be happy for me to send through your details and have them call you? **If yes to only 1:** The questions are related to the welfare of people who call in from the flood and cyclone areas. It appears that you have not noticed a large number of changes; you only answered yes to one question. If you find that this is causing you concern either now or in the future, we suggest you contact your GP to further discuss this. Thank you for answering these questions. **If no to all:** The questions are related to the welfare of people who call in from flood and cyclone affected areas. It appears you have not noticed any changes in your welfare, but please talk to your GP or call us back if you get concerned in the future. Thank you for answering these questions.	

Abbreviations: 13HEALTH, 24-hour Queensland, Australia phone service; GP, General Practitioner.

This paper describes and evaluates the 13HEALTH component of the post-disaster mental health response program. The evaluation assesses: (1) the effectiveness of opportunistic screening via a general health 24-hour call line; (2) whether the 13HEALTH screening program was cost-effective; (3) whether the severity of the floods or cyclones influenced the number of calls and the likelihood of callers being personally affected by the disaster and at-risk of emotional distress, despite the call not being related to mental health concerns; and (4) whether a positive score using opportunistic screening resulted in agreement by the caller for referral to the SMHP.

## Method

The paper is a descriptive, retrospective study of non-mental health seeking callers to a 24-hour State-wide health line (13HEALTH). Ethics approval was granted by Metro South Health (Woolloongabba, Queensland, Australia) Human Centre for Health Research Ethics Committee (HREC/14/QPAH/472) – a retrospective evaluation of the outcomes of State-wide disaster mental health programs established and delivered following the cyclones and floods of 2010-2011; and Queensland University of Technology (Brisbane, Queensland, Australia; Ethics approval number 1500000016).

The calls to 13HEALTH were identified and recorded for each HHS area, except for the Children’s HHS, which provides a State-wide tertiary service. Metro North and Metro South HHS data were combined, as one SMHP provided the Brisbane-based services. The screening process occurred with the consent of each caller. Those who consented and were directly affected or resided in a disaster-affected region were screened for symptoms related to the impact of trauma.

The clinician identified whether the call related to the individual caller or another household member. Those calling about a child or young person were asked whether they agreed to answer a single question about the event’s impact on the child. The inquiry focused on emotional or behavioral changes since the disaster. If the answer indicated changes, the child was offered a referral for assessment by a clinician from the local SMHP.

The 13HEALTH clinician administered the Primary Care-Posttraumatic Stress Scale (PC-PTSD)^25^ to adults who consented to the assessment protocol. The PC-PTSD Scale is a four-item screening measure (Table 1) commonly used within primary care and community-based out-patient clinics. A 13HEALTH clinician administered the questions. The PC-PTSD Scale has a test-retest reliability of 0.83 and adequate sensitivity and specificity (>80%).^26^ Each response item, using a Likert Scale, assesses either re-experiencing a traumatic event, emotional numbing, avoidance, and hyper-arousal phenomena, key features of posttraumatic stress disorder (PTSD). Those scoring more than two were provided information on the emotional impact of disasters and services that could assist, including the nearest SMHP team. With the caller’s consent, a referral was emailed to a local SMHP. The local SMHP staff contacted the individual within 24-72 hours. Callers scoring two or below were provided information about services and advised to seek assistance should they notice increasing distress.

## Results

From January 2012 through December 2012, 13HEALTH received 205,064 calls. A number of the calls (n = 891) related to a child or young person under 18. The number of calls per HSS varied from 101 to 103,559 over the twelve months (Table 2). The distribution of callers reflected the Queensland population density by HHS region. The Queensland population in 2011 was 4,332,739 with 1,169,781 (26.99% of the population) aged between 0-19.^27^

**Table 2.** Number of Callers to 13HEALTH per Hospital and Health Service

Catchment Area	Number of 13Health Callers	Percent (%)
Cairns	8,295	4.05%
Cape York	101	0.05%
Central	9,729	4.74%
Central West	142	0.07%
Darling Downs	10,448	5.09%
Mackay	6,120	2.98%
Metro North + Metro South	103,559	50.50%
Gold Coast	17,118	8.35%
Sunshine Coast	13,535	6.60%
South West	349	0.17%
Torres Strait	32	0.02%
Townsville (incl Mt Isa)	10,913	5.32%
West Moreton	14,311	6.98%
Wide Bay	10,412	5.08%
total	205,064	100.00%

Abbreviation: 13HEALTH, 24-hour Queensland, Australia phone service.

The number of callers progressing through each stage of the screening procedure was recorded and summarized at the HSS and State-wide levels. The percentage of consumers passing through each screening stage, culminating in referral to the SMHP, was calculated (Table 3).

**Table 3.** Record of Calls and Assessment of Calls January-December 2012

	Queensland 4,332,739 (ABS Data 2011)	Percent (%)
Total Calls to 13HEALTH	205,064	4.73% of state population
Number of callers identified as from flood/cyclone affected areas	19,708	9.61% of all calls
Number of callers identified as personally affected by flood/cyclone	7315	37.12% (of those living in the affected area)
Number of adults screened for PTSD	3883	53.08% (of those personally affected
Number of adults at risk of PTSD	907	23.36% (of those screened)
Number of adults referred	700	77.18% (of those who score >2 on PC-PTSD scale)
Number of children/toddlers screened for PTSD	891	0.08% of the population under 18 (approximately)^a^
Number of children at risk of PTSD	290	32.55% of those screened
Number of children referred	207	71.38% of those considered at risk.

Abbreviations: 13HEALTH, 24-hour Queensland, Australia phone service; PTSD, posttraumatic stress disorder.

a Australian Bureau Statistics age range is 0-19. Data in the paper refers to ages 1-18.

**Figure 1.**
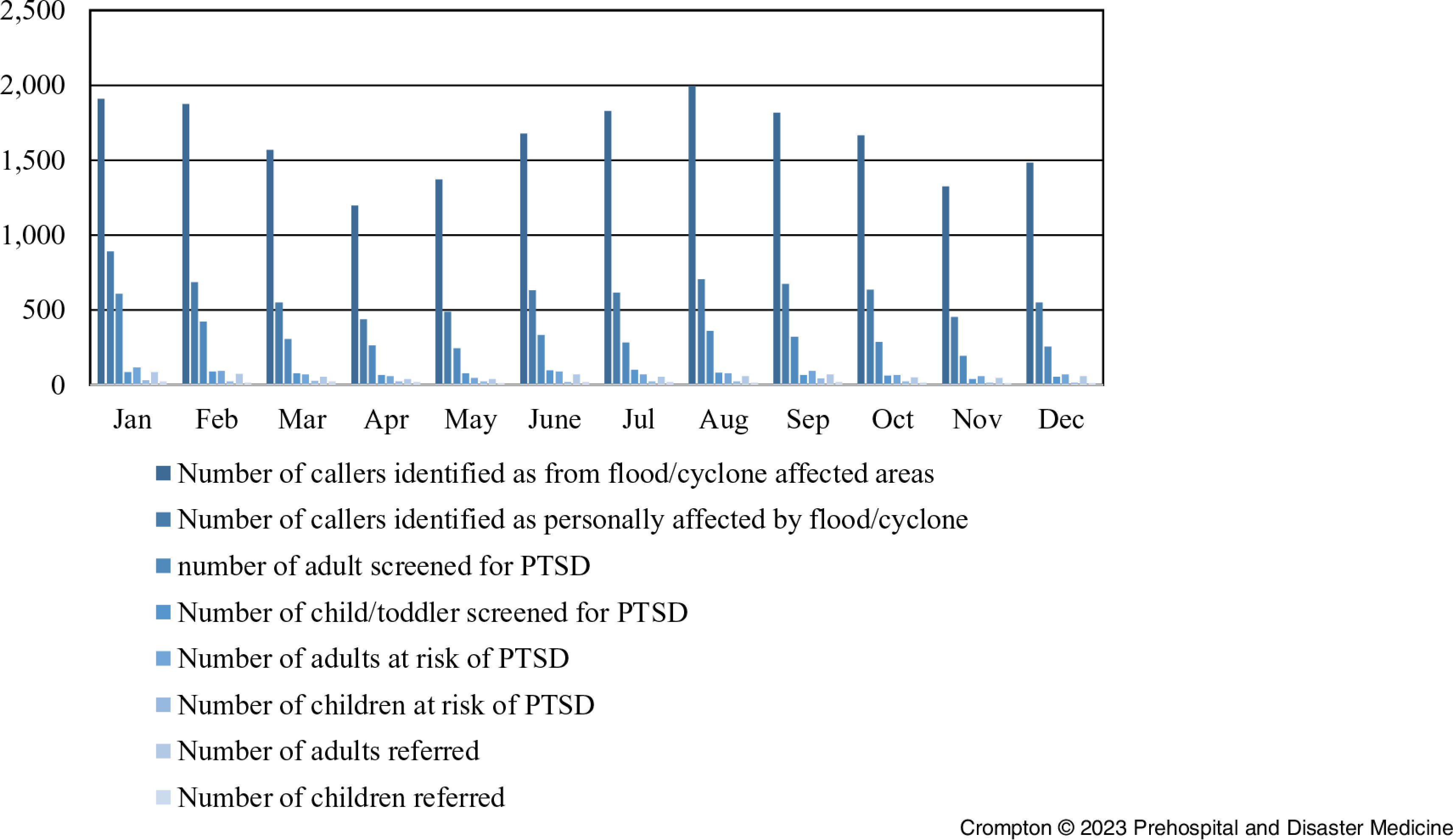
State-Wide 13HEALTH Screening. Abbreviations: 13HEALTH, 24-hour Queensland, Australia phone service; PTSD, posttraumatic stress disorder.

There were 19,708 callers who resided in a flood or cyclone-affected area. The callers represented 0.48% of the Queensland population. From this group, it was ascertained that 7,315 (aged over 18) were personally affected, representing approximately 0.23% of the Queensland population over 18 (ABS Census data age range is 0-19). Of adults personally affected, 3,883 (53%) agreed to complete the PC-PTSD Scale, and 907 scored more than two (approximately 0.03% of the Queensland adult population; Table 3 and Figure 1). The data indicated 891 calls related to those under 18 (approximately 0.08% of the Queensland population aged under 18). The single-item question showed that 290 may have experienced emotional or behavioral changes following the floods and cyclones.

The percentage of adult callers (53%) who agreed to complete the PC-PTSD Scale contrasts with those who consented to answering the under-18 survey question (22.9%). The number of adult callers assessed by the PC-PTSD and scored more than two was 907, with 77.17% agreeing to a referral to the specialized intervention program. Of the 290 under 18 considered at-risk, 71.38% accepted referral to a SMHP.

The descriptive analysis identified that the regions most affected by the floods and cyclones were more likely to have callers to 13HEALTH. The regional and rural areas of Cairns, Central Queensland, the Darling Downs, South-West, Townsville, West Moreton, and Wide Bay HHS were areas most affected by the cyclones and flooding, representing 4.05%, 4.74%, 5.09%, 0.17%, 5.32%, 6.98%, and 5.08%, respectively, of all a region’s callers to 13HEALTH. The call numbers approximated the population distribution of Queensland for the HHS. Data analysis indicated that callers from these regions were more likely to report living in an area affected by the natural disasters. Cairns, Central Queensland, the Darling Downs, South-West, West Moreton, and Wide Bay exposure rates were 9.74%, 6.07%, 10.93%, 0.42%, 12.76%, 12.19%, and 7.43%, respectively. While the south-east corner of Queensland represents approximately 50% of the State’s population data, only 4.1% of the Brisbane major statistical region households were affected.^28^ The number from Brisbane who reported they lived in a flood area was 28.45% (Table 4). Within these regions, the number who said they were personally affected was also elevated compared to the number of callers from areas less affected by the disasters (Table 5).

**Table 4.** Number of Callers from a Flood or Cyclone-Affected Area

Catchment Area	Number of 13Health Callers	Percent (%)
Cairns	1,920	9.74%
Cape York	11	0.06%
Central	1,321	6.70%
Central West	16	0.08%
Darling Downs	2,154	10.93%
Mackay	707	3.59%
Metro North + Metro South	5,607	28.45%
Gold Coast	316	1.60%
Sunshine Coast	1,188	6.03%
South West	83	0.42%
Torres Strait	2	0.01%
Townsville (incl Mt Isa)	2,515	12.76%
West Moreton	2,403	12.19%
Wide Bay	1,465	7.43%

Abbreviation: 13HEALTH, 24-hour Queensland, Australia phone service.

**Table 5.** Identified as Personally Affected by either the Floods or Cyclones

Catchment Area	Number of 13HEALTH Callers	Percent (%)
Cairns	784	10.72%
Cape York	4	0.05%
Central	402	5.50%
Central West	7	0.10%
Darling Downs	801	10.95%
Mackay	234	3.20%
Metro	1,997	27.30%
Metro (Gold Coast)	102	1.39%
Metro (Sunshine Coast)	430	5.88%
South West	35	0.48%
Torres Strait	1	0.01%
Townsville (incl Mt Isa)	1,100	15.04%
West Moreton	915	12.51%
Wide Bay	503	6.88%

Abbreviation: 13HEALTH, 24-hour Queensland, Australia phone service.

Analysis of the data related to the program found that self-reported location concerning the flood or cyclone and personal exposure, in conjunction with the PC-PTSD Scale screening tool, was an efficient, low-cost population screening strategy (Table 2 and Table 3). The 13HEALTH-screening program cost $5.66 (AU) per PC-PTSD Scale screen. The total cost of the screening program in 2012 was $53,284 (AU).

The screening protocol readily identified those living in regions affected by the disaster, with the numbers affected consistent with the level of inundation or cyclone damage for the various regions (Table 4 and Table 5).

## Discussion

This study indicates that opportunistic screening via a State-wide general health line provides a cost-effective mechanism for screening callers who contacted 13HEALTH due to physical health concerns. The screening distinguished those living in affected areas from individuals personally affected by the cyclones and flooding. The cost of the program in 2012 was $53,284 (AU). The equivalent cost in 2022 Australian dollars for screening the same number of people is approximately $65,073 (AU).^29^ The screening costs included identifying those who lived in a natural disaster-affected region and whether the person was personally affected, the provision of information regarding the psychological impact of the events, administering the PC-PTSD Scale, referral to the SMHP, and provision of information regarding community resources. Although the program was cost-efficient and covered the entire State with 24-hour per-day access, it is evident that an individual must have telephone access and personally contact the service for an assessment to occur. In disaster-affected areas, infrastructure damage or property loss can limit access to landlines, mobile telephone services, or computers, particularly in the early phase of the disaster response.

The analysis reveals that adults contacting the service for a general health reason were likely to indicate they were personally affected, but less often agreed to participate in the PC-PTSD Scale screening (53%). Callers were even more unlikely to answer a question related to the impact of the natural disasters on a child or young person (22.9%). The effectiveness of the program as a public health screening strategy that may reduce the likelihood of delayed intervention and unmet community mental health needs is demonstrated by the disparity between the initial helpline, which had five callers in six months, and the numbers identified as personally affected by the floods and cyclones using opportunistic screening (n = 7,315).

The initial program required people to call a “hotline” to discuss mental health concerns. In contrast, the 13HEALTH line received calls from those experiencing physical health problems and/or psychological distress, although this paper only evaluates those calling due to physical health concerns. The inter-relationship between pre- and post-disaster physical health and mental health outcomes across the lifespan^30^ and by gender has been identified in several papers. Lowe, et al (2016 and 2019) found that following the 2010 Deepwater Horizon Spill (DHS), those involved in the physical response to the event were more likely to experience medical-related symptoms. There was an increased risk of anxiety symptoms, including PTSD phenomena and major depression. Significantly, those exposed to the DHS and Hurricane Katrina in 2005 were at greater risk of experiencing physical health symptoms.^31,32^ A systematic review of south and south-east Asian women impacted by natural disasters noted the relationship between adverse physical and mental health outcomes.^33^ The importance of gender is exemplified by the caregiver roles adopted by women in many societies, the increased risk of intimate partner violence (IPV) following a disaster, and the recognized association between IPV and the increased likelihood of adverse physical health and mental health outcomes.^34–37^ Failure to address the relationship between post-disaster physical health and adverse psychological outcomes and to provide a strategy to address this aspect increases the likelihood of unmet mental health needs and delayed presentation and treatment.

The descriptive analysis identified that the number of callers to 13HEALTH across Queensland in 2012 reflected the population distribution across the HHS. The regions most affected by the floods and cyclones, when compared to the less affected areas, were more likely to have callers concerned about a physical disorder. These regions also reported more people personally affected by the floods and cyclones. The percentage of callers personally affected varied from 1.3 to 2.3 times greater in regional Queensland. The likelihood of callers with a physical concern being from areas with high rates of personal exposure to the floods or cyclones is particularly evident in Brisbane.^38^ Although the number of affected households represented only 4.1% of the total number of Brisbane households,^28^ around 28% of callers stated they were personally affected. This study highlights the number of people who, while calling about their physical health, also were experiencing mental health concerns. These findings reflect Paranjothy’s conclusions (2011) that linked the level of flooding with the risk of adverse mental health outcomes. The 13HEALTH study data indicate there were more personally affected callers with greater levels of psychological distress from the most disaster-affected regions.^14^

Although gender was not identified as part of the intake call record, unpublished data indicate that 75% of those referred to the SMHP for assessment identified as female, suggesting that most callers (n = 7,081) identifying as personally affected were women. Given the literature reports on IPV following a disaster, the potential and value of opportunistic screening is further highlighted by the 2012 data. The callers, therefore, represent a group at significant risk for exposure to IPV and delayed diagnosis and treatment.

From the group (aged 18+) who indicated they were personally affected (n = 7,315) and were personally affected, 891 scored more than two on the PC-PTSD Scale and were offered referral to the SMHP. Despite a screening score more than two on the PC-PTSD Scale, only 77.17% of adults agreed to a specialized disaster treatment program referral. For those under 18 and screened as at risk, the percentage of parents or carers who agreed to a referral to a treatment program was slightly lower (71.38%). The progression from identifying risk for a mental disorder to participating in treatment is challenged by several factors. Stigma remains a crucial barrier to entry into psychological therapies, as are the negative perceptions of mental health care.^39,40^ A study of unemployed persons found mental health literacy and the structure of treatment programs negatively influenced treatment participation.^41^ Studies involving farming communities note the issue of stigma, concerns regarding anonymity, the role of stoicism, and distrust of health professionals.^42,43^ Other studies report the perception of needing to be self-reliant remains a significant barrier to seeking care.^44^ In relation to those under 18, a United States study found that most children in need of mental health care were not receiving the appropriate interventions due to a failure to recognize the symptoms, limitations of parental knowledge about mental illness, and stigma.^45^ Despite the increased use of digital health interventions, there is evidence that digital services do not enhance parental help-seeking, mental health literacy, or service utilization.^46^

The challenge in responding to natural disasters such as the recent events in northern New South Wales and Southeast Queensland include the regional geography, the multiple episodes of flooding, the number of people affected, and access to clinical services. These aspects are similar to the issues that confronted Queensland in 2010-2011. The Queensland response was developed to address the risk of unmet needs by enhancing access to psychosocial and community support services and SMHP. The Plan was part of a broad State-wide program that included economic, social, and infrastructure responses.^23^ The disaster response plan was informed by the Australian disaster management guidelines and the disaster guidelines from other countries.^47–49^

The size of the disaster and the geography of Queensland provided a challenge in terms of access to disaster mental health services, ensuring community knowledge of these services, and addressing the unmet mental health needs of people affected by the floods and cyclones. The State-wide 24-hour 13HEALTH triage line provided information regarding local community and mental health services and information about psychosocial problems that may arise post-disaster while enabling the instruction of opportunistic screening of callers affected by the events. The importance of linking services and using well-identified programs such as 13HEALTH is emphasized by the small number (n = 5) who contacted a specialist mental health support line established six months after the disasters.

While the 13HEALTH screening instrument focused on phenomena related to PTSD, there is evidence the PC-PTSD Scale screening questions were also likely to screen for depression. The association between disaster exposure and the development of depression is well-recognized, with a recent study finding that a post-disaster positive response to the Brief Trauma Questionnaire (BTQ) was associated with an increased likelihood of depression.^50^ Although the opportunistic screening data derived from the 24-hour access line highlight this strategy’s success, the previous self-initiated mental health hotline’s lack of success suggests several approaches should be adopted to address unmet needs. One method would be establishing an outreach model focusing on areas most affected by the natural disaster. This strategy would also utilize predictive weather and disaster modelling to identify the most at-risk regions. A targeted program that includes screening questions that focus on personal experiences related to disaster exposure and brief clinical screening questionnaires such as the PC-PTSD Scale, the Kessler-10, or General Health Questionnaire-12 delivered by digital, telephone, and face-to-face strategies has the potential to amplify the effectiveness of a public health outreach program.^51^

However, even a more targeted call center program with a broader range of mental health assessments will be challenged by factors such as the reluctance of people to acknowledge and communicate the adverse impact of the disaster to health care services. This reluctance is exemplified by the response of health care workers (HCWs) who, during the coronavirus disease 2019 (COVID-19) pandemic, demonstrated a disinclination to use the pandemic helpline established to support HCWs.^52,53^

Prior to the introduction of a “hot line” following Hurricane Katrina, only one call center effectiveness study had been undertaken.^54^ Despite the increased use and uptake of telehealth and other electronic services offered by helplines, there remain gaps in service provision and evaluation, the responsiveness of these services, barriers to access, and limitations concerning integration with the broader disaster response.^55–58^

With the development of *smartphones,* the potential of telemedicine has expanded. Mobile applications (apps) can facilitate multiple functions, including psychoeducation, assessment, interventions, and both asynchronous and synchronous connection to clinicians. Similar to the role of helplines, a rate-limiting step is a desire by the affected person to engage with the app. Following disasters, access to a reliable, useable smartphone may be compromised.^59,60^ A small pilot study (n = 11) of a PTSD app, while noting the acceptability of the app, also found that it was only slightly to moderately helpful. Almost one-half of the subjects did not use the app as intended.^61^ The COVID-19 pandemic has encouraged research regarding digital interventions. The authors of a Canadian study identified 31 apps and 114 web-based programs. They found marked variability in terms of the technology platforms, cost, and focus of the programs and minimal assessment of their efficacy. This review also noted the lack of equity with respect to first nation people, language, and culture.^62^ A qualitative study of *PTSD Coach Australia* pointed out that while clinicians perceived the app as a useful monitor of symptoms between treatment sessions, they also identified it as prone to technical problems. The study also identified difficulties with the user-interface functionality.^63^

Although there has been advancement in technology and further development of digital and mobile apps since the 2010-2011 Queensland floods and cyclones, the findings of this study remain relevant. Providing an age-appropriate point of community access, a source of information regarding mental health care, and the ability to connect with the appropriate services that meet the cultural and language needs of the individual and the community is critical. Importantly, the program should be integrated with the overall disaster response.^55,62^

## Limitations

This study focuses on PTSD symptoms rather than on a broader array of measures that address the other psychological and behavioral outcomes that may occur following exposure to traumatic events such as a natural disaster. The absence of measures that assess symptoms related to depression, other anxiety disorders, substance use, and whether there has been a change in interpersonal relationships biases the study towards PTSD-type symptoms. Consequently, there is a risk of under-estimating the level of psychological need within the community and a failure to address the unmet need of those experiencing mental health and behavior changes. The absence of information pertaining to the gender of callers may further influence the degree of unmet community mental health care. At the same time, the lack of detail concerning why people refused to be screened is relevant to the broader understanding of the utility of opportunistic screening and how the screening methodology and the engagement of those screened can be improved.

## Strengths

The study has several strengths, including the opportunity to compare a mental-health-specific person-initiated call line versus a naturalistic opportunistic screening strategy using a general health call line. The number of callers to 13HEALTH is significant, as is the number screened and referred to the SMHP (Table 2). The presence of data that links the severity of flooding or cyclone exposure to the number of callers who rang due to physical problems, and subsequently identified as being at risk for psychological distress, supports the findings of other studies that have emphasized the link between the severity of flooding and mental health outcomes. The study also highlights the degree of unmet need following a disaster.

## Conclusion

Evaluation of the 13HEALTH screening program implemented following the natural disasters in Queensland (2010-2011) demonstrates opportunistic population screening can identify at-risk individuals who may require specialist mental health care. The screening protocol in this program adopted a brief assessment system that, in future disasters, could be improved by including depression and anxiety questionnaires. The study highlights that callers to a health line may have unrecognized mental health problems related to the natural disaster.

The evaluation indicates disaster severity modelling could enable the establishment of targeted screening programs for regions at greater risk. Such an approach would support a screening program focusing on areas defined by flood height, building damage, or rates of injury or death. A more focused approach supports identifying those at risk of a mental disorder and directing psychosocial interventions to the most vulnerable. The screening program demonstrated the economic and clinical benefits of opportunistic screening of incoming calls to a general health call center. Using a general health call center also provides an opportunity to screen individuals for exposure to the disaster. It acknowledges that for many, the psychological impact may not be recognized or considered and addresses the hesitancy people may have in seeking help to address their mental health.

Importantly, there is a need to establish the program early in the disaster response and recovery phases, and for the screening strategy to be integrated with the broader disaster response and community communication strategies. Including an outreach, post-disaster screening program that focuses on the most at-risk groups could further enhance post-disaster screening for individuals at risk of mental health disorders. However, there remains a need for post-disaster evaluations of screening strategies.
